# Maternal–fetal stress and DNA methylation signatures in neonatal saliva: an epigenome-wide association study

**DOI:** 10.1186/s13148-022-01310-x

**Published:** 2022-07-14

**Authors:** Ritika Sharma, Martin G. Frasch, Camila Zelgert, Peter Zimmermann, Bibiana Fabre, Rory Wilson, Melanie Waldenberger, James W. MacDonald, Theo K. Bammler, Silvia M. Lobmaier, Marta C. Antonelli

**Affiliations:** 1 Department of Obstetrics and Gynecology, Klinikum Rechts der Isar, Technical University of Munich, Munich, Germany; 2 Department of Obstetrics and Gynecology and Center On Human Development and Disability (CHDD), University of Washington, Seattle, WA USA; 3 Research Unit of Molecular Epidemiology, Institute of Epidemiology, Helmholtz Zentrum Munich, Munich, Germany; 4 Instituto de Fisiopatología y Bioquímica Clínica (INFIBIOC), Facultad de Farmacia y Bioquímica, Universidad de Buenos Aires, Buenos Aires, Argentina; 5 Department of Environmental and Occupational Health Sciences, University of Washington, Seattle, WA USA; 6 Instituto de Biología Celular y Neurociencia “Prof. E. De Robertis”, Facultad de Medicina, Universidad de Buenos Aires, Buenos Aires, Argentina

**Keywords:** Pregnancy, Prenatal stress, Perceived stress, Biomarkers, Newborn saliva, DNA methylation, Cortisol, Epigenetics, EWAS

## Abstract

**Background:**

Maternal stress before, during and after pregnancy has profound effects on the development and lifelong function of the infant’s neurocognitive development. We hypothesized that the programming of the central nervous system (CNS), hypothalamic–pituitary–adrenal (HPA) axis and autonomic nervous system (ANS) induced by prenatal stress (PS) is reflected in electrophysiological and epigenetic biomarkers. In this study, we aimed to find noninvasive epigenetic biomarkers of PS in the newborn salivary DNA.

**Results:**

A total of 728 pregnant women were screened for stress exposure using Cohen Perceived Stress Scale (PSS), 164 women were enrolled, and 114 dyads were analyzed. Prenatal Distress Questionnaire (PDQ) was also administered to assess specific pregnancy worries. Transabdominal fetal electrocardiograms (taECG) were recorded to derive coupling between maternal and fetal heart rates resulting in a ‘Fetal Stress Index’ (FSI). Upon delivery, we collected maternal hair strands for cortisol measurements and newborn’s saliva for epigenetic analyses. DNA was extracted from saliva samples, and DNA methylation was measured using EPIC BeadChip array (850 k CpG sites). Linear regression was used to identify associations between PSS/PDQ/FSI/Cortisol and DNA methylation. We found epigenome-wide significant associations for 5 CpG with PDQ and cortisol at FDR < 5%. Three CpGs were annotated to genes (Illumina Gene annotation file): ***YAP1****, ****TOMM20*** and ***CSMD1***, and two CpGs were located approximately lay at 50 kb from ***SSBP4*** and ***SCAMP1***. In addition, two differentiated methylation regions (DMR) related to maternal stress measures PDQ and cortisol were found: ***DAXX*** and ***ARL4D***.

**Conclusions:**

Genes annotated to these CpGs were found to be involved in secretion and transportation, nuclear signaling, Hippo signaling pathways, apoptosis, intracellular trafficking and neuronal signaling. Moreover, some CpGs are annotated to genes related to autism, post-traumatic stress disorder (PTSD) and schizophrenia. However, our results should be viewed as hypothesis generating until replicated in a larger sample. Early assessment of such noninvasive PS biomarkers will allow timelier detection of babies at risk and a more effective allocation of resources for early intervention programs to improve child development. A biomarker-guided early intervention strategy is the first step in the prevention of future health problems, reducing their personal and societal impact.

**Supplementary Information:**

The online version contains supplementary material available at 10.1186/s13148-022-01310-x.

## Background

Compelling evidence from both animal and human studies indicates that adversities in the perinatal environment significantly increase the risk for developing neurocognitive disorders later in life [1–10].

Human studies provide substantial evidence that maternal stress during the gestational period (namely PS) can lead to behavioral, cognitive and temperamental disorders in the infant, increasing child morbidity and neurological dysfunction. For example, maternal psychosocial stress (general and specific stress and anxiety) increases the risk for the growing infant to develop disorders such as attention-deficit hyperactivity disorder (ADHD) [10], autism spectrum disorders (ASD) [11] and sleep disturbance that can result in depression and other psychiatric disorders. Severe PS is associated with increased cortisol response after a behavioral challenge paradigm in young adults [12]. Moreover, several studies have indicated that PS and glucocorticoid exposure may reprogram the cardiovascular system, including aberrations in cardiac and kidney development [13].

It is important to understand the mediators that connect the mother’ stress with the fetus. In this regard, Rakers et al. [8] proposed that the causal pathway lies not only via cortisol but also includes catecholamines, reactive oxygen species, cytokines, serotonin/tryptophan and maternal microbiota. The stress response system has been traditionally linked to the hypothalamic–pituitary–adrenal (HPA) axis that is responsible for the production and secretion of corticosteroids under basal and stressed conditions [14]. However, the autonomic nervous system (ANS) also plays a key role through its rapid (within seconds) activation enabling fine adjustments of the target organs. This aspect has been often neglected. In this regard, Monk et al. [15] have shown that the fetal ANS is very perceptive of maternal anxiety. This study shows that during women’s recovery following a stress-eliciting task, fetal heart rate (fHR, a biomarker of ANS) changed in association with the mother’s acute cardiovascular activity [15]. In addition, fHR sensitivity to a stimulus reflects emerging individual differences in the development of ANS [16, 17].

Responding to environmental factors, these stress-mediating pathways are assumed to leave permanent epigenetic signatures that may affect the neurobehavioral outcomes of the child. In fact, it has been shown that the epigenome is vulnerable to external exposures during the early prenatal development, a crucial period when intense programming of gene expression is taking place [18, 19]. Among these mechanisms, DNA methylation is recognized as the most well-characterized epigenetic signature.

While some knowledge has been gained linking the scale of epigenetic organization of phenotypical information with the psychological behavior [20, 21], the connection between the epigenetic signatures of adversity and the scale of biophysical organization of human integrative physiology has remained unexplored. This is of great interest because biophysical behavior, such as the properties of ANS describable noninvasively by mathematical HRV analyses, is highly accessible, now more than ever, with the rise of the wearable technologies and remote health monitoring [22]. As such, linking these scales of physiological organization may aid in early detection of unhealthy developmental trajectories and timely intervention while also providing a more mechanistic multi-scale framework for understanding these complex relationships [7]. With the present ‘FELICITy’ study, we aimed to address this knowledge gap.

First, we hypothesized that early epigenetic signatures of PS are detectable in neonatal saliva. This is important because the detection of such epigenetic signatures, ideally at birth, will help to detect ‘at-risk children’ who can benefit from early stimulation programs and follow-up [23]. Second, we hypothesized that late gestation biophysical alterations in mother–fetus dyads due to PS are reflected in the neonatal epigenetic marks observed at birth. To test these hypotheses, we devised the FELICITy study to obtain a combination of noninvasive multimodal physiological measures of PS.

We recruited a cohort of third trimester pregnant women screened for exposure to chronic psychosocial stress during pregnancy. The biophysical signature of the PS exposure has been validated during late gestation using noninvasive feto-maternal transabdominal electrocardiogram (taECG). This approach yielded fetal stress index (FSI), a joint maternal–fetal biomarker of PS [24]. At the same time, we also acquired quantitative psychological maternal chronic stress scores (details in Results and Methods) [24]. Using maternal behavioral data and the biophysical FSI assessment on the one hand and the epigenetic analysis of the newborn’s saliva on the other hand, we aimed to validate the presence of linkages between the mother–fetus behavior and the neonatal saliva’s epigenetic modifications. To test for persistence of such multimodal multi-scale linkages we are currently following up this cohort for a second time point at two years of age. Here, we report the existence of such anticipated linkages at birth. The study represents a first report of a well-controlled, prospective study to investigate epigenome-wide methylation changes in > 850.000 loci in newborn saliva samples in association with behavioral and biophysical maternal–fetal stress measures.

## Results

We performed an Epigenome-Wide Association Study (EWAS) with the Illumina MethylationEPIC BeadChip array, which includes around 850,000-methylation loci in saliva samples obtained from newborns at the time of delivery. We examined the association between prenatal exposure to maternal psychosocial stress and offspring genome-wide saliva methylation using four statistical approaches in association with four complementary maternal and fetal stress measures. The stress measures are: 1) the PSS-10 questionnaire, accounting for the perceived stress of a mother during the third trimester; 2) the PDQ questionnaire accounting for the specific worries related to pregnancy such as pain during labor and delivery, personal appearance after delivery and baby’s health; 3) maternal hair cortisol levels (integrated cortisol levels in hair reflecting three prior months of stress exposure) accounting for the chronic activity of the HPA stress response system of the mother during the third trimester and 4) the FSI, a biophysical ANS biomarker for stress, which accounts for fetal ANS reactivity to maternal heart beat during pregnancy.

### Cohort characteristics

Of the 728 subjects who returned the questionnaires, the socio-demographic characteristics of the participating mothers and their offspring (n = 114) are shown in Table 1. The average age of the mother at study entry was 34 years. Most of the mothers in this study were Caucasians, married and having a university degree. Pregnancies were mostly planned, and 72% of mothers had vaginal deliveries. The perceived stress measures (PSS and PDQ scores) were moderately correlated (Spearman R2 = 0.537; *p* value = 6.952e-10).

**Table 1:** Baseline characteristics of study population (n = 114, mother–newborn pairs), FELICITy study

Characteristics	n = 114
*Maternal characteristics—Baseline*	
Age of mother at study entry, years	34.61 (± 4.52)
Gestational age at screening, weeks	34.07 [33.18, 34.96]
Gestational age at inclusion, weeks	36.71 [35.32, 37.54]
Score PSS	17.00 [9.00, 22.00]
Score PDQ	10.50 [6.25, 16.75]
BMI at study entry, kg/m^2^	27.61 [25.20, 30.42]
BMI pregestational, kg/m^2^	21.82 [20.30, 24.96]
Ethnicity, Caucasians/Europeans	106 (92.98)
Married, yes	85 (74.6)
University degree, yes	80 (70.2)
Household income > 5000€/month, Yes	53 (46.5)
Working status at screening, working	4 (3.5)
Multiparity, Yes	85 (74.6)
Planned pregnancy, Yes	92 (81.4)
Cesarean delivery, Yes	28 (24.6)
Smoking, Yes	7 (6.1)
IVF / ICSI, Yes	10 (8.8)
Gestational diabetes, Yes	11 (9.6)
Autoimmune disease, Yes	15 (13.2)
Cortisol in maternal hair, pg/mg	97.00 [58.00, 161.00]
*Infant characteristics—Perinatal outcome*	
FSI	0.15 [−0.28, 0.60]
Gestational age at birth, weeks	39.71 [38.86, 40.57]
Gender, female	52 (45.6)
Birthweight, grams	3544.96 (± 431.78)

Data are mean (SD) using chi-square test, mean (interquartile) using Wilcox. test or n (%) using Fisher’s test. All the continuous variables are shown as [mean (SD)], [median(range)] and the categorical variables as n (%)

*PSS* Cohen Perceived Stress Scale, *PDQ* Prenatal Distress Questionnaire, *BMI* body mass index; *ICSI* intracytoplasmic sperm injection; *IVF* in vitro fertilization; *FSI* Fetal Stress Index

### Differentially methylated positions (DMPs)

Each CpG site was separately tested for association with exposure to stress (PSS, PDQ, Cortisol and FSI), and separate linear regression models were run, unless otherwise specified. All the models were adjusted as specified in the Methods section.

#### DNA methylation and stress measures

##### DNA Methylation and PSS score

**Fig. 1:**
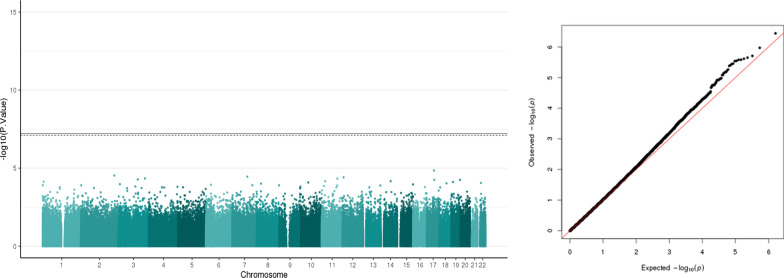
Manhattan plot and Q–Q plot of salivary DNA methylation associated with PSS. Manhattan plots of salivary DNA methylation associated with PSS. The x-axis represents the genomic loci of the individual CpGs and the y-axis represents the –log10 (p value). Black line: Bonferroni threshold (p = 6.183879e-08) and the dotted line: Multiple testing correction threshold (FDR < 0.05) has been added to the plot. There are no CpGs that cross the significance threshold. Quantile–quantile (QQ) plot shows the expected and the observed quantiles

PSS score (the continuous variable) was used for the association analysis. We did not identify any differentially methylated sites in relation to PSS score. Figure 1 shows the Manhattan plot and the Q–Q plot. Table 2 shows the top four hits for the association with no significant findings.

**Table 2:** CpG sites associated with stress measures in DNA methylation analysis

Stress measure	Probe	Coef^d^	P.Value^e^	FDR^f^	chr^g^	Illumina gene annotation	Genes within 50 kb of associated CpG
**PSS**^**a**^	cg17478679	−0.2	3.59E-07	0.25	chr17	KPNB1	CRHR1
	cg22124215	−0.1	1.07E-06	0.25	Chr2	MARCH4	DIRC3
	cg06195987	0.52	1.96E-06	0.25	Chr7	NA	LMTK2
	cg15426815	0.31	2.24E-06	0.25	Chr12	MIR200C	C1S
**PDQ**^**b**^	**cg06542869**	**0.02**	**4.62E-08**	**0.03**	**chr11**	**YAP1**	**YAP1**
	cg22861369	0.02	2.30E-07	0.08	chr5	PDLIM4	SLC22A4
	cg01629131	0.03	3.31E-07	0.08	chr20	NA	RP11, RP1
	cg03105159	0.01	7.18E-07	0.11	chr2	ALKAL2	ALKAL2, FAM105B
**Cortisol**	**cg11409463**	**0.003**	**2.87E-09**	**0.002**	**chr5**	**NA**	**SCAMP1**
	**cg20905655**	**0.004**	**1.16E-07**	**0.03**	**chr19**	**NA**	**SSBP4;**
	**cg25252839**	**0.002**	**1.24E-07**	**0.03**	**chr1**	**TOMM20**	**SNORA14B**
	**cg05306225**	**−0.002**	**2.08E-07**	**0.04**	**chr8**	**CSMD1**	**CSMD1**
**FSI**^**c**^	cg13547817	−0.39	8.85E-08	0.07	chr9	ERP44	ERP44; INVS
	cg07642729	−0.35	2.81E-06	0.49	chr8	ASB15	-
	cg24795351	−0.34	3.79E-06	0.49	chr8	PREX2	PREX2
	cg16692227	−0.21	3.80E-06	0.49	chr14	SAMD12	SAMD12

The table shows top four CpGs from the EWAS that are associated with the respective stress measures. Marked in **bold** are significant

^a^Cohen Perceived Stress Scale

^b^Prenatal Distress Questionnaire

^c^Fetal Stress Index

^d^Regression coefficients from the statistical model

^e^Significance from the statistical model

^f^False discovery rate

^g^Chromosome

NA: Not available

##### DNA methylation and PDQ score

**Fig. 2:**
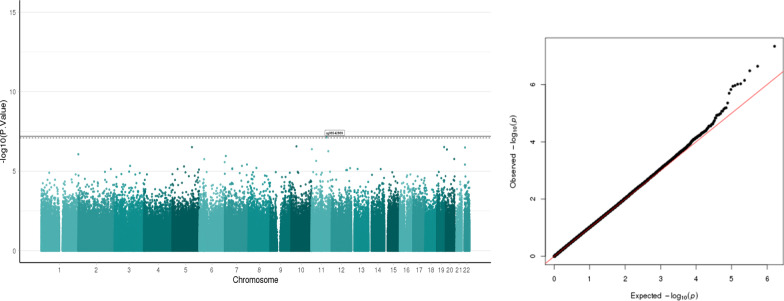
Manhattan plot and Q–Q plot of the association between PDQ and salivary DNA methylation. Manhattan plots of salivary DNA methylation associated with PDQ. The x-axis represents the genomic loci of the individual CpGs and the y-axis represents the –log10 (p value). Black line: Bonferroni threshold (p = 6.183879e-08) and the dotted line: Multiple testing correction threshold (FDR < 0.05) has been added to the plot. CpGs that cross the FDR threshold are marked in the Manhattan plot. There is 1 CpG that crosses the significance threshold. Quantile–quantile (QQ) plot shows the expected and the observed quantiles

The association analysis with PDQ scores yielded one CpG (cg06542869, p = 4.62E-08) (Fig. 2) achieving FDR < 0.05. This site has a positive direction of effect, and it is located in the body of the protein coding gene *YAP1* (Yes1-regulated transcription factor) present in chromosome 11 (Table 2). The regression coefficients and values for the next three nonsignificant hits for this association are reported in Table 2. The Q–Q plot shown in Fig. 2 is corrected for inflation which has a lambda value of 1. 04. The uncorrected vs the corrected Q–Q plot is shown in Additional File 1: Fig. S1.

##### DNA methylation and cortisol

**Fig. 3:**
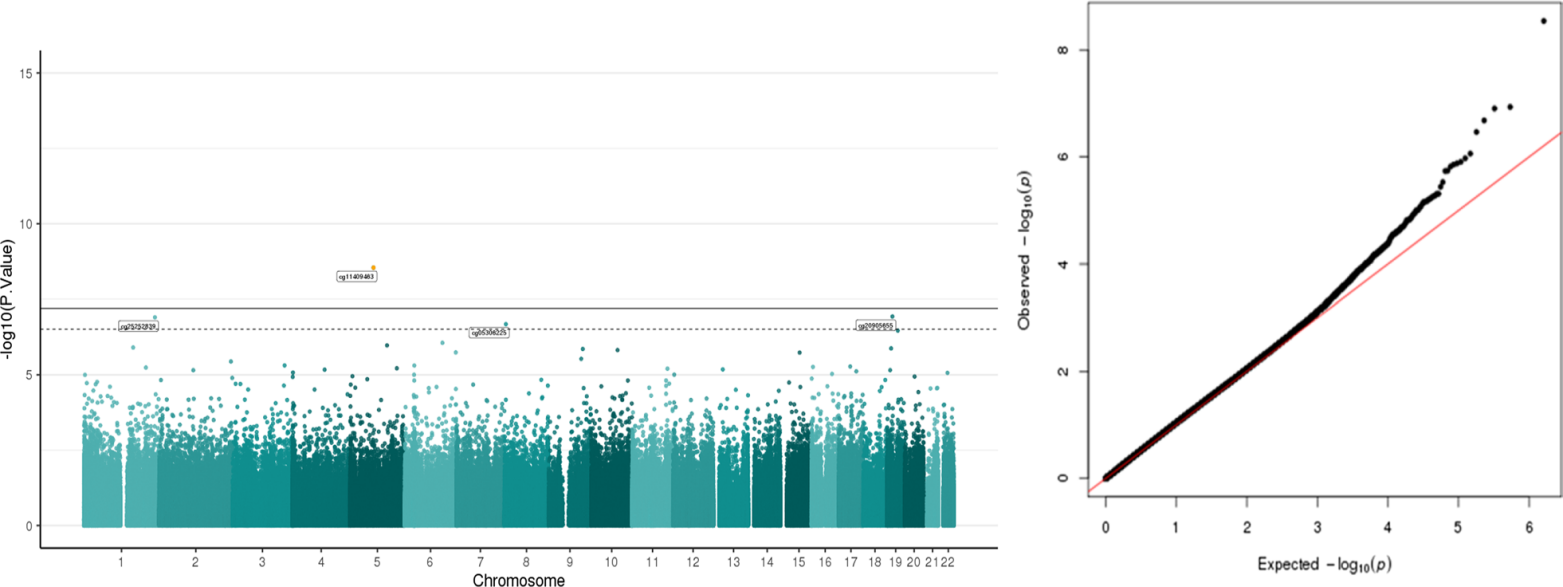
Manhattan plot and Q–Q plot of the association between cortisol and salivary DNA methylation. The lambda value for the Q–Q plot is 1.08. Manhattan plots of salivary DNA methylation associated with cortisol. The x-axis represents the genomic loci of the individual CpGs and the y-axis represents the –log10 (p value). Black line: Bonferroni threshold (p = 6.183879e-08) and the dotted line: Multiple testing correction threshold (FDR < 0.05) have been added to the plot. CpGs that cross the FDR threshold are marked in the Manhattan plot. There are four CpGs that cross the FDR multiple correction threshold. Quantile–quantile (QQ) plot shows the expected and the observed quantiles

We identified stronger associations with cortisol compared to other stress variables (Fig. 3, Table 2). Four CpG sites were identified after controlling for multiple testing using FDR < 0.05 (cg11409463, cg20905655, cg25252839 and cg05306225). The top hit was cg11409463, located on chromosome 5 but did not annotate to any gene via the Illumina gene annotation file. A look up on University of California Santa Cruz (UCSC) browser showed that the nearest genes within 50 kb distance to this CpG site are *SCAMP1* (Secretory Carrier Membrane Protein 1) and ***AP3B1*** (Adaptor Related Protein Complex 3 Subunit Beta 1). This CpG site also overlaps with several transcription factors from the AP-1 family. The second hit was cg20905655 (p = 1.16E-07) located on chromosome 19 which did not annotate to any gene via Illumina platform. According to the UCSC genome browser, the nearest genes within the 50 kb region are ***SSBP4*** (single-stranded DNA binding protein 4) (Castro et al., 2002) and ***LRRC25*** (leucine-rich repeat containing 25). The third hit was ***TOMM20*** (translocase of outer mitochondrial membrane 20) (cg25252839, p = 1.24E-07) located on chromosome 1. All the CpG sites had a positive direction of association except for the fourth hit, cg05306225, which annotates for the gene ***CSMD1*** (CUB and Sushi multiple domains 1) located on chromosome 8 and encoding for Q96PZ7-CSMD1_HUMAN (CUB and Sushi domain-containing protein 1). Inspection of quantile–quantile (QQ) plot did not show evidence for inflation or bias (Fig. 3; lambda = 1.08).

##### DNA methylation and FSI

**Fig. 4:**
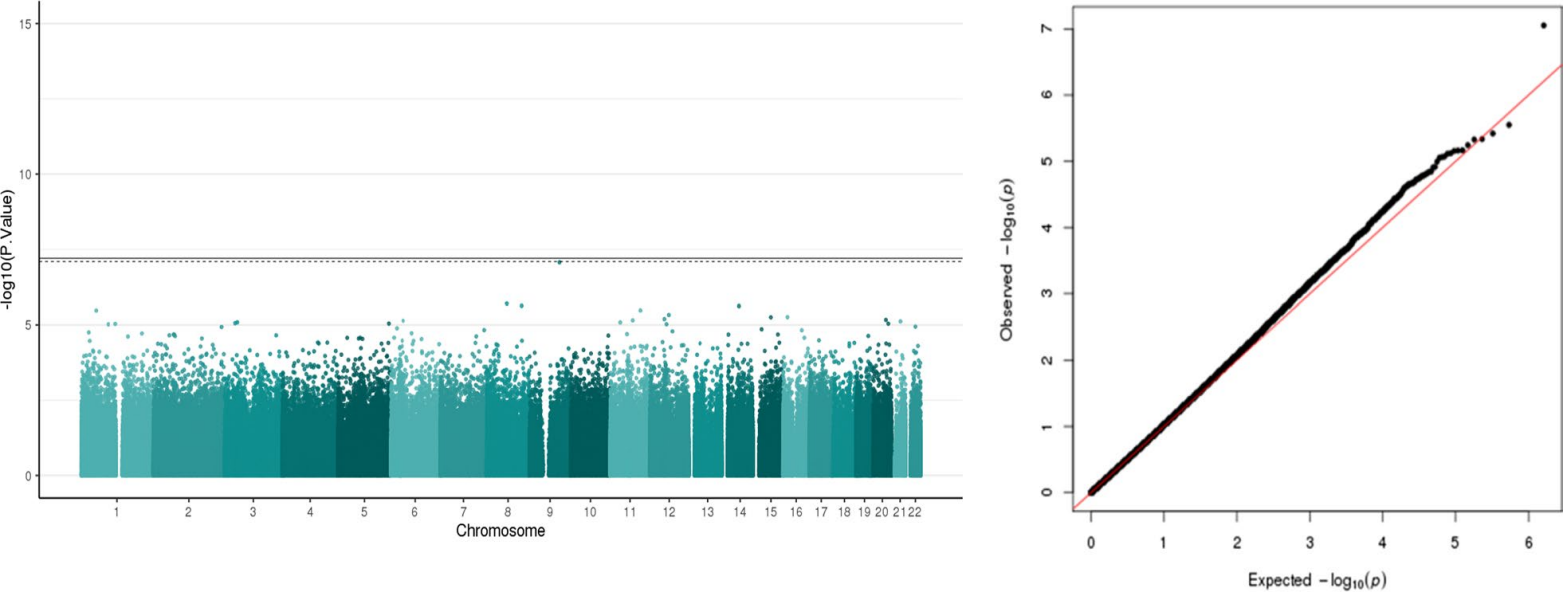
Manhattan plot and Q–Q plot of the association between FSI and salivary DNA methylation. Manhattan plots of salivary DNA methylation associated with FSI (Fetal Stress Index). The x-axis represents the genomic loci of the individual CpGs and the y-axis represents the –log10 (p value). Black line: Bonferroni threshold (p = 6.183879e-08) and the dotted line: Multiple testing correction threshold (FDR < 0.05) has been added to the plot. CpGs that cross the FDR threshold are marked in the Manhattan plot. There are no DMPs that cross the Bonferroni correction threshold. Quantile–quantile (Q–Q) plot shows the expected and the observed quantiles and has a lambda 1.01

There were no DMPs that survived the correction for multiple testing when the association was performed with FSI, the biophysical biomarker of PS exposure. Of interest, the top hit, CpG (cg13547817, p = 8.51E-08) with an FDR: 0.06, very close to the threshold, mapped to the gene ***ERP44*** (Endoplasmic reticulum protein 44) on chromosome 9, which is a protein coding gene whose related pathways are the Innate immune system and translational control. Table 2 shows the top four hits from the FSI association analysis and Fig. 4 shows the Manhattan plot and the Q–Q plot.

#### Sex specificity analysis

The CpG-by-sex interaction analysis did not reveal any significant differences between sexes for the associations (Table 3).

**Table 3:** Epigenome-wide results of the Interaction analysis between gender and stress measures

Stress measures	Probe	Coef^d^	*p* Value^e^	FDR^f^	Chr^g^	Illumina gene annotation
PSS^a^	cg09723184	0.03	6.23E-06	0.87	chr8	FBXO43
	cg27293447	−0.04	7.25E-06	0.87	chr2	LOC102800447
PDQ^b^	cg03756940	−0.05	1.10E-07	0.08	chr2	NA
	cg00008621	−0.04	2.06E-07	0.08	chr14	HIF1A
Cortisol	cg18197866	0.003	2.55E-07	0.16	chr12	PXN
	cg20460797	−0.006	4.17E-07	0.16	chr4	NSG1
FSI^c^	cg24715106	0.54	2.51E-07	0.2	chr11	AQP11
	cg23782719	−0.42	2.54E-06	0.56	chr6	RNF182

The table shows top two CpGs from the EWAS of the interaction analysis that are associated with the stress measures

*NA* Not available

^a^Cohen Perceived Stress Scale

^b^Prenatal Distress Questionnaire

^c^Fetal Stress Index

^d^Regression coefficients from the statistical model

^e^Significance from the statistical model

^f^False discovery rate

^g^Chromosome

### Exploratory analysis of DMPs

In a second layer of analysis, the network interactions between the proteins encoded by the genes that were annotated to significant DMPs were analyzed using STRING-db, a database and software application enabling an semi-unsupervised statistical network analysis of known and predicted protein–protein interactions as well as their physical and functional interaction networks based on computational predictions, i.e., enrichment [25]. Unique URL for the resulting analysis is as follows:

https://version-11-5.string-db.org/cgi/network?taskId=bMnf9hIgSYrT&sessionId=bl9Pry04KzyB.

**Fig. 5:**
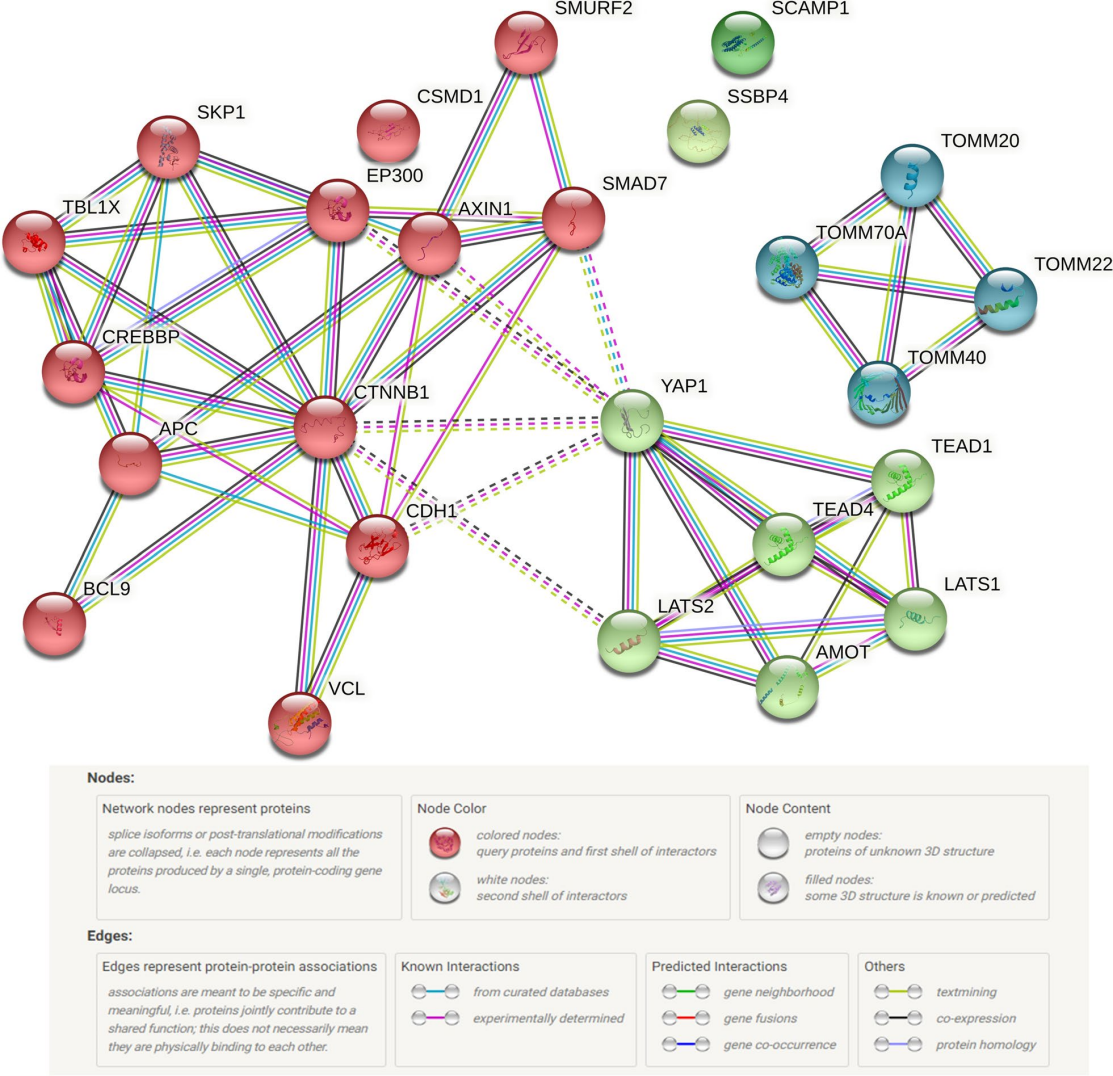
Network plot of significant hits from the EWAS analysis. STRING-Db network analysis for significant hits from the association for PDQ and cortisol. Protein–protein interaction (PPI) enrichment p value: 3.47e-06. PPI legend by string-db.org. The permanent link is: https://version-11-5.string-db.org/cgi/network?taskId=bvfqNrZYaHe6&sessionId=bjK7XvqNxMXe

The protein–protein interaction (PPI) enrichment *p* value for this network is 3.47e -06. The top three biological processes identified were 1) Hippo signaling pathway, 2) regulation of canonical Wnt signaling pathway and 3) cell–cell junction assembly. Figure 5 shows YAP1 interacting with several proteins of the Hippo signaling pathway and the ß-catenin signaling pathways-CTNNB1 (Catenin Beta 1). YAP appears also directly related to TEAD1 and TEAD4 since YAP/TAZ are transcriptional coregulators and partners of the TEAD family transcription factors.

The Kyoto Encyclopedia of Genes and Genomes (KEGG) pathways map molecular objects such as genes and proteins to molecular interactions or relations. The top pathway identified from the KEGG pathways was the Hippo signaling pathway.

As the next step, we used the SFARI gene database [26] to extract information for the genes annotated to CpGs specific to ASD [27]. SFARI gene is a database centered on genes involved in autism and has up-to-date information on all human genes associated with ASD. Of all the genes looked up in this database [26], only *CSMD1* appeared with a score of three indicating strong relevance to ASD (gene.sfari.org/database/human-gene/*CSMD1*).

### Differentially methylated regions (DMRs)

DMRs are genomic regions that have consistently different DNA methylation across multiple adjacent CpGs [28]. The DMRs mapped to or near the genes that are enriched for the biological process of the regulation of sequence-specific DNA binding transcription factor activity suggest that these genes are involved in regulation of gene expression. Regional analysis identified associations with maternal stress measures (PDQ and Cortisol). All DMRs identified by DMRcate as well as the DMPs overlapped with the DMRs identified by comb-p. Results are shown in Table 4.

**Table 4:** Differentially methylated regions (DMRs) in salivary DNA associated with stress measures in FELICITy study

Stress measures	Chr^b^	Start (bp^c^)	End(bp)	CpGs^d^	p value^e^	Sidak P^f^	Gene
**PDQ**^**a**^	**6**	**33,288,180**	**33,288,600**	**8**	**0.000166981**	**8.67E-08**	**DAXX**
**Cortisol**	**17**	**41,476,044**	**41,476,457**	**11**	**6.50E-10**	**1.27E-06**	**ARL4D**

Marked in **bold** are significant

^a^Prenatal Distress Questionnaire

^b^Chromosome

^c^Physical position (basepair)

^d^Number of probes in the region

^e^Statistical significance

^f^*p* of Sidak multiple testing correction

DMRcate identified two DMRs related to maternal stress measures PDQ and cortisol. One DMR associated with PDQ was found to be located in the ***DAXX*** locus (death-associated protein 6) on chromosome 6. The other DMR, associated with cortisol, was found to be located in the ***ARL4D*** (ADP-ribosylation factor 4D) on chromosome 17.

## Discussion

Most of the maternal stress studies in the past have been limited to targeted DNA methylation analyses in candidate genes [29, 30]. It is only in the recent years that epigenome-wide studies of DNA methylation have gained popularity allowing to evaluate locus-specific methylation across the entire genome [31–34]. These different approaches have been recently summarized in two reviews and a meta-analysis [18, 35, 36]. When analyzing these studies, a general conclusion on what type of epigenetic signature is observed in prenatally stressed infants is difficult to draw since many methodological differences are still observed in terms of the type and timing of the prenatal insult studied, the age of the child and the tissue employed to detect DNA methylation. This makes comparison among studies very difficult leading to inconclusive evidence on the association between PS and DNA methylation in the neonate. To bridge this gap, we have examined the association between psychological, molecular and biophysical maternal–fetal stress measures and the genome-wide methylation profile in newborn saliva. Our findings validate the hypothesis that PS biomarkers are associated with epigenome–wide DNA methylation in newborn saliva across multiple CpG sites, in particular, those relevant to neuronal, immune and endocrine homeostasis.

### Maternal stress measures and DNA methylation patterns

In our study, PDQ, but not PSS, and cortisol showed a significant association with five CpG sites. Out of these five CpGs, three were annotated to ***YAP1****, ****TOMM20*** and ***CSMD1;*** two CpGs were not annotated to any gene but lay within 50 kb of ***SSBP4***, ***SCAMP1*** and ***LRRC25***. We discuss the functional implications of these associations in the following paragraphs.

***YAP1*** and its related protein WWdomain-containing transcription regulator 1 (WWTR1; also known as TAZ) (YAP/TAZ) are the main effectors of the Hippo signaling pathway [37]. This evolutionarily conserved signaling cascade regulates cell proliferation, stemness, organ size control and regeneration. Its dysregulation has been associated with multiple forms of cancers, the immunity response and cardiovascular diseases [37, 38]. Although widely expressed in several tissues, *YAP* is selectively expressed in astrocytes and neural stem cells in the mouse developing brain and its deletion causes reactive astrogliosis and astrocyte-driven microglial activation [39]. Moreover, Passaro et al. [40] demonstrated that the transient downregulation of YAP in mouse embryonic stem cells disrupts cellular homeostasis altering the ability to differentiate properly. In our study, the hypermethylated CpG cg06542869 annotated to *YAP1* is associated with specific pregnancy worries (PDQ score). The functional consequence of the hypermethylation of one single CpG site in the open sea of the *YAP1* gene is highly speculative without evaluating the translated protein. However, it has been demonstrated that the methylation of one single CpG can impact on the methylation levels of neighboring CpG sites [41]. Assuming that hypermethylation is generally associated with transcriptional silencing of genes, the modification of methylation status of the *YAP1* gene might potentially lead to alterations in cell proliferation, cell differentiation and astrogliosis. In fact, the network analysis of the protein encoded by *YAP1* using STRING-db showed an interaction with several proteins of the Hippo signaling pathway such as the TEAD family of transcription proteins. The phosphorylation and inhibition of YAP/TAZ activate the Hippo pathway limiting tissue growth and cell proliferation. Upon dephosphorylation, YAP/TAZ translocate to the nucleus, binding to TEAD and inducing transcriptional programs related to cell proliferation, survival and migration [37].

***TOMM20*** (translocase of outer mitochondrial membrane 20) is involved in glucose/energy metabolism and deubiquitination. Together, TOMM22 functions as a transit peptide receptor at the surface of the mitochondrial outer membrane and facilitates the movement of preproteins [42, 43]. Diseases associated with TOMM20 include Optic Atrophy 1 and 11. Our results show that the hypermethylated CpG site cg25252839 is associated with cortisol levels and annotates to *TOMM20*.

***The CSMD1*** gene has been proposed to have brain specificity since it encodes a cell adhesion molecule highly expressed in membrane-associated proteins in the CNS, with almost no detection in other tissues [44]. The CSMD1 protein is related to immune function playing a crucial role in regulating complement activation and inflammation in the developing brain [44, 45] and may also play a role in growth cone function [46]. The CSMD1 protein is predominantly expressed in neurons mainly in the cerebral cortex and the hippocampus and has been involved in brain circuits development, neurotransmission, axon guidance, regeneration and plasticity [44]. ***CSMD1*** protein coding gene has been previously associated with autism spectrum disorders (ASD) [47, 48]. Corroborating the above statement, ***CSMD1*** also appears on the SFARI database listing genes associated with ASD. It scored as level 3, meaning there is suggestive evidence from significant but non-replicated association studies. Moreover, ***CSMD1*** has been associated with post-traumatic stress disorder [49, 50], schizophrenia [44, 45, 51, 52], and bipolar disorders [53].

In our study, we found that the hypo-methylated CpG cg05306225 annotates for the gene ***CSMD1*** and is associated with high maternal cortisol levels. Although it is difficult to predict the functional consequences of this single site hypomethylation as mentioned above, it is interesting to observe that the probable destabilization of the methylation status of flanking CpGs mentioned before, is in a gene with high brain specificity and associated with several neuropsychiatric disorders. In particular, the association of this gene with ASD refers back to several reports showing that the risk for ASD is linked to PS [11, 54].

The two other CpGs that were significantly associated with cortisol levels but are not annotated to any gene are cg11409463 and cg20905655, both hypermethylated. The nearest gene to the CpG site cg11409463 is ***SCAMP1*** whose protein is involved in secretion and transportation. Diseases associated with this gene include Childhood Kidney Cell Carcinoma and Branchiootorenal Syndrome 1. This same CpG site overlaps with several transcription factor binding sites from the AP-1 family (the Jun, the Fos and ATF-2 subfamily) such as JUNB, FOS, SETDB1, ATF3, CBX3, TRIM28, ZNF143. The AP-1 family is responsible for cell growth, differentiation [55] and apoptosis [56]. The nearest genes to CpG site cg20905655 are ***SSBP4*** and ***LRRC25****,* the latter related to autophagic degradation. So far, not much is known about the functional role of ***SSB4*** and its relation to stress yet.

Of interest, cortisol-associated methylation disbalances in several genes found in neonatal saliva suggest that the transplacental barrier might be impaired and abnormally permeable to steroid hormones. In fact, it has been described that the metabolizing enzymes that lay within trophoblasts and protect the fetus from overexposure to glucocorticoids, are sensitive to maternal stress [57, 58]. For example, the glucocorticoid-inactivating enzyme, 11β-hydroxysteroid dehydrogenase type-2 (11βHSD2), showed a reduced placental expression in relation to maternal anxiety and depressed mood in humans [59, 60]. The reduced placental expression of 11βHSD2 will potentially lead to a fetal glucocorticoid overexposure affecting developmental events such as fetal growth restriction, altered HPA axis development, impaired offspring brain function, permanent changes in the expression of specific transcription factors and early development of proliferative neural precursors [57, 61]. Our observation that the newborn saliva shows cortisol-associated epigenetic changes in genes related to energy metabolism, cell differentiation and function of the developing brain might be highlighting that one of the underlying mechanisms linking maternal stress with childhood outcomes is through transplacental mediated methylation disbalances in specific genes, among other mechanisms, such as transcriptional regulation of placental gene expression as suggested by Aushev et al. [62].

To expand the search of epigenetic signatures associated with stress measures during pregnancy we considered DMRs. Two DMRs were detected, one associated with PDQ (***DAXX***) and the other to cortisol (***ARL4D***). ***DAXX*** gene encodes for a protein that resides in multiple locations in the nucleus and cytoplasm. Pathways related to Daxx are apoptosis and survival caspase cascade as well as TGF-β signaling pathways [63]. Diseases associated with ***DAXX*** include Gastric neuroendocrine neoplasm, intellectual disability and alpha-thalassemia. Interestingly, ***ATRX*** gene which has been previously linked with ASD, interacts with ***DAXX*** in histone chaperone complex and influences DNA methylation [64–66]. Moreover, ***DAXX*** is known to be an extended Class II, non-antigen binding ***HLA*** (human leukocyte antigen) gene associated with autoimmune diseases that interacts with death receptor Fas related to ASD [67]. ***ARL4D*** belongs to ADP-ribosylation factors (ARFs), members of the Ras family of small GTPases, involved in membrane transport, membrane lipid modifications and maintenance of organelle integrity [68]. Interestingly, the transcription of Arl4d was found to be consistently regulated by glucocorticoids such as cortisol [69]. So far, not much is known about its function, but it has been shown that Arl4D is involved in neurite growth [70], adipogenesis [71] and actin remodeling [72]. In adult mice, *Arl4d* is expressed in neocortical layer 1 and hippocampus, mostly in cortical interneurons (CIN), whose loss or alteration have been related to neurological disorders such as autism, schizophrenia, and epilepsy [73]. Interestingly, both DMRs are directly or indirectly related to neurological disorders such as ASD. To the best of our knowledge, this is the first report showing significant DMRs in the PS context in newborn saliva samples. Previously, Drzymalla et al. [74] have identified DMRs related to maternal stress but using cord blood.

Previous studies employing EPIC array on neonatal tissues in association with maternal stress and/or anxiety are limited to one study by Kallak et al. [75]. These authors investigated DNA methylation in cord blood of newborns exposed to maternal depression and anxiety. They found two DMPs: one upstream of the ATP Binding Cassette Subfamily F Member 1 gene (***ABCF1***) and the other upstream of Homo sapiens integrator complex subunit 10 gene (***INTS10***). Although the maternal stress model is different from ours, it is interesting to note that ***ABCF1*** was previously associated with ASD in a multi-omics data analysis [76]. Other comparable studies employing Illumina Infinium 450 BeadChip found mismatching results when studying DNA methylation in infant tissues in relation to maternal stress. Rijlaarsdam et al. [77] showed no associations between PS exposure and neonatal cord blood DNA methylation, whereas Wikenius et al. [78], studying maternal depressive symptoms, found no significant association with 6-week infant’s saliva DNA methylation. In contrast, Non et al. [33] reported the identification of CpGs located in a cluster of genes related to transcription, translation and cell division processes in cord blood of neonates exposed to non-medicated depression or anxiety.

To summarize these results, we conclude that these genes have been related to several regulatory processes of tissue and cellular homeostasis that, when disturbed, can elicit a stress response [79]. Moreover, dysregulation of the expression of ***CSMD1*** and ***YAP1*** has been related to disorders of the immune system as well as of the central and autonomic nervous systems.

### Biophysical signature of chronic stress in mother–fetus dyad and DNA methylation

No significant CpG sites were observed in association with FSI. This may be either due to insufficient study power or reflect an underlying mechanism. Namely, it is possible that regardless of the non-specific chronic stress perceived by the mother (PSS) and the ANS response of the fetus (FSI), what most impacts the fetal epigenetic profile is the stress generated by specific worries related to pregnancy (captured by PDQ) and the associated high circulating levels of cortisol that is crossing the maternal–fetal placental barrier and impacting the fetal physiology on the scale of epigenome. It is possible that FSI is not the appropriate biophysical correlate of epigenome-level alterations due to PS. In future studies, to investigate this relationship further we intend to analyze in more depth the relationships between the neonatal epigenome and the biophysical features of ANS derived from maternal and fetal HRV.

### Strengths and limitations

In the present study, we report the findings of the largest prospectively followed cohort of its kind to date. Several strengths are to be highlighted. Firstly, saliva cells are easy and noninvasive to obtain in newborns. Even though epigenetic changes like DNA methylation are cell and tissue-specific, some CpG sites show cross-tissue relevance. Changes in peripheral tissues such as saliva could serve as potential biomarkers for disease risk while also giving an advantage of being noninvasively obtainable. Since the primary organ affected by stress is not available in human studies and postmortem brain tissue samples cannot capture the fluid state of the epigenome [80], more accessible samples such as saliva and blood are often used as substitutes. Binder and colleagues showed that saliva reflects better DNA methylation patterns of the brain than methylation in blood, highlighting that saliva is the sample medium of choice for epigenetic studies of psychiatric traits, especially in small children [81].

Secondly, we believe that our study’s findings can be generalized to the population of pregnant women in most clinics, as this study includes mothers experiencing typical daily stress situations rather than extreme stress exposures.

Limitations to our study are as follows. Our study has a relatively small sample size, which makes identifying subtle differences in methylation difficult. Originally, we powered the study based on the primary outcome in this project: a difference in the child’s mental developmental index at 24 months of age between infants from stressed mothers and controls. With this in mind, assuming a relevant difference in means of 5 with a SD of 10 [82] (alpha 5% and power [1-beta] 80%), we needed to include 63 stressed mothers in our analyses. To account for 15% dropout, we aimed at for 75 stressed women. Given the figures in the literature [83], we expected around 10% screen-positives on the anxiety screener. As we show in Additional File 1: Fig. S2, 2000 patients received the questionnaires and 728 patients were screened upon returning the questionnaires.

Since there is no other available study with cohorts of pregnant women and newborn saliva samples obtained, we have not yet been able to verify our findings in an independent cohort. The only way to validate that one EWAS study compares to another is to use the same psychological tests in a similar population. Comparison with other studies is difficult since we have used a more narrowly defined chronic stress paradigm, saliva medium, and a newer Infinium array that may have together increased our chances in discovering meaningful CpG associations. However, the novel findings of DMPs and DMRs related to these stress measures in newborn saliva should be considered as hypothesis-generating and requires further validation in larger cohorts.

Assessing the DNA methylation levels as soon as the baby is born in association with four stress measures shows the impact of maternal stress on epigenetic marks during the fetal life. However, to serve as early neurodevelopmental biomarkers these marks have to be related to the corresponding neurodevelopmental appraisals. Since epigenetic marks are not fixed at birth and methylation patterns change with age, we are presently carrying out a longitudinal study in this cohort. The DNA methylation status at 2 years of age will allow us to detect the epigenetic drift defined as the difference in the DNA methylation status over time [78]. Moreover, the 2 years of time point will allow us to test for an association with the neurodevelopmental outcome showing the influence of the environment during the first two years of life on the epigenetic traits and whether the present early neonatal epigenetic differences can serve as biomarkers for early interventions to help restore optimal neurodevelopmental trajectories [23].

## Conclusions

In this study, we identified novel associations between newborn epigenome-wide methylation levels measured noninvasively in saliva and chronic psychosocial stress experienced by the mother during pregnancy. The epigenetic changes are mostly related to genes involved in secretion and transportation, nuclear signaling, Hippo signaling pathways, apoptosis, intracellular trafficking and neuronal signaling. Most strikingly, we found that both DMP (such as ***CSMD1*)** and DMRs (***DAXX and ARL4D***) are annotated to genes related to neurological disorders such as ASD, PTSD and schizophrenia, pointing out to the potential risk of these children to suffer from these disorders.

Taken together, our findings demonstrate that newborns exposed to chronic stress during gestation show DNA methylation signatures related to neuronal, immune and endocrine homeostases.

## Materials and methods

### Study design

Women with singleton pregnancies, between ages 18 and 45 in their third trimester (at least 28-week gestation) were recruited at the Department of Obstetrics and Gynecology at ‘Klinikum rechts der Isar’ of the Technische Universität München (TUM). Exclusion criteria were serious placental alterations, fetal malformations and maternal severe illness during pregnancy or use of recreational drugs [24]. Between June 2016 and July 2019, 164 women were recruited. Due to methodological problems with saliva sampling, methylation data was available for 114 subjects.

### Measures

#### Exposure: Maternal stress during pregnancy

Stress can be assessed using general self-report instruments designed for pregnant women and these maybe more predictive of the perinatal outcome than generic stress inventories and able to assess pregnancy-related stress. We used psychosocial stress assessment instrument (PSS and PDQ) and also measured stress as chronically accumulated cortisol using maternal hair samples.

##### (a) Psychosocial stress assessment

Maternal psychosocial stress was measured using the validated German version of Cohen Perceived Stress Scale (PSS-10) [84]. PSS-10 is a widely used psychological instrument to measure non-specific perceived chronic stress and measures the degree to which a situation in a person's life is appraised as stressful. It has been validated in German-speaking population and is a quick tool for screening chronic stress among prospective subjects [85]. In addition, the validated German version of the Prenatal Distress Questionnaire (PDQ) was also administered to the participants to assess specific pregnancy worries and concerns [24, 86–89]. PSS score and PDQ score were correlated using the Spearman method in R studio.

##### (b) Hair cortisol assessment

After delivery, maternal hair strands (~ 3 mm diameter) were collected from the posterior vertex region on the head as close to the scalp as possible. The hair samples were sent to the Department of Biochemistry (Endocrinology section) of the Faculty of Pharmacy and Biochemistry (University of Buenos Aires, Argentina) for cortisol measurement using an automated chemiluminescent immunoassay. This method was validated, and putative confounders such as dye, washing or dandruff shampoo were shown to not interfere with cortisol measurements [90] Based on the hair growth rate of 1 cm per month, the 3 cm long hair segment reflects the integrated hormone secretion over the three-month period prior to sampling. The cortisol was extracted and measured according to Iglesias et al. [91]. This procedure has been validated with the standard method of mass spectrometry and was patented by University of Buenos Aires [90].

### Exposure: fetal stress

#### Fetal stress assessment

The detailed fetal assessment is described elsewhere [24]. In brief, bivariate PRSA (bPRSA) was used to assess the coupling between maternal (mHR) and fHR resulting in Fetal Stress Index (FSI). The fHR was measured by taECG. Fetal ECG extraction algorithm SAVER [92] was applied to detect the fetal R-peaks and the maternal R-peaks in the taECG separately. With the fetal and maternal R-peaks, the fetal and maternal RR interval time series were obtained. Mean fHR and mean maternal heart rate were calculated. Generally, bPRSA identifies and quantifies the relationship between two simultaneously recorded signals. Here, the two signals are mHR as the trigger signal and fHR as the target signal. FSI measures the response of fHR to decreases in mHR.

### Outcome: DNAm measurement from newborn saliva

#### Sample and data acquisition

##### Newborn saliva sampling

Immediately after delivery, the midwife obtained the newborn saliva/buccal sample by gently rubbing the gums on both sides with the sponge of the Oracollect-DNA kit (DNA Genotek, Canada) and stored it at room temperature. Throughout the manuscript and for ease of reading, we have referred as ‘saliva’ to the sample containing both the saliva fluid plus leukocytes and squamous epithelial cells from the oral cavity [93].

##### DNA extraction

DNA was extracted from 1 ml saliva samples using the PrepIT kit (DNA Genotek). A 1 µl aliquot of the extracted samples was checked on 0.8% Agarose gel and measured via NanoDrop for quality. A 2 µl aliquot of DNA extract from the samples was used to run PCR to check for the sex of the newborn.

##### Illumina MethylationEPIC BeadChip array

Bisulfite conversion of DNA and processing of methylation arrays were accomplished in collaboration with the Institute of Epidemiology (Complex diseases group) at Helmholtz Zentrum Munich. For each sample, 500 ng of the extracted salivary DNA was treated with sodium bisulfite using the EZ–DNA methylation kit following the manufacturer’s protocol. DNAm was assessed using the Illumina Infinium Human MethylationEPIC BeadChip array (Illumina Inc., San Diego, CA, USA) according to the manufacturer’s instructions. This array measures over 850,000 loci at a single-nucleotide resolution. The BeadChip includes probe types of two different chemistries: [1] Type I probes, in which two different probe types interrogate each CpG site, one which targets methylated DNA and one that targets unmethylated DNA. [2] Type II probes binding to the nucleotide just before the target site, and create a single base extension of G or A complementary to the methylated C or unmethylated T.

### Data processing

#### Quality control

The arrays were scanned using an Illumina iScan reader and processed using GenomeStudio software (Illumina, Inc.). The raw data (idat files) were imported into R using the Bioconductor minfi package [94] and CpGs that have below-background expression levels in more than six samples were filtered out. Concordance between the reported sex and methylation predicted sex was confirmed. Probes with common single-nucleotide polymorphism (SNPs) near the methylation binding site were identified and filtered out. To simplify the analysis, probes were restricted to those on autosomal chromosomes. The remaining probes were background-corrected using the out-of-bound probes [95], and normalized using a functional normalization procedure, which uses two principal components of a set of control probes in order to remove technical variability [96]. The β-values (proportion of methylated probes at each CpG) were then converted to M-values (logit base 2 of the β-values), which were used for all linear modeling. To account for unobserved variability or potential batch effects, models were additionally adjusted with three surrogate variables (SV) that were generated from the M-values, using the Bioconductor ‘SVA’ package [97]. The surrogate variables were used as covariates in the statistical analysis. The final analysis included 808,554 probes. Linear regression was used to examine the associations of each CpG site with stress measures. Probes were considered significantly differentially methylated at a false discovery rate (FDR) < 0.05 [98].

##### Covariates

**Fig. 6:**
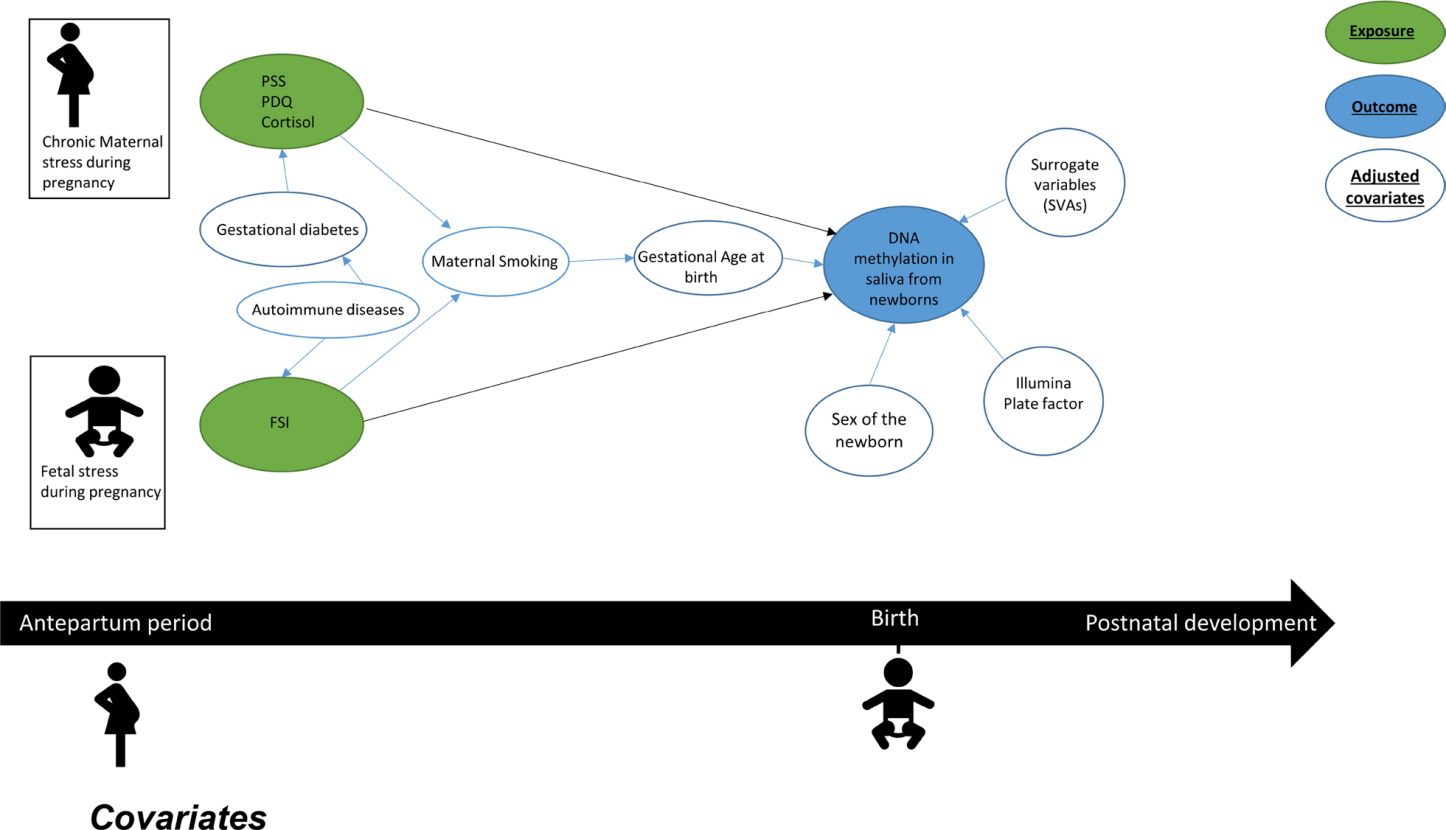
Direct acyclic graph (DAG) displaying the hypothesized associations between maternal and fetal stress and infant salivatory DNA methylation

Figure 6 shows the proposed DAG that displays the covariates used in our analysis. We included covariates such as newborn sex, gestational age at birth, maternal smoking, autoimmune diseases and gestational diabetes. The sex of the newborn was obtained from the clinical history of the patient. Gestational age was calculated from the first day of the woman’s last menstrual cycle to the date of delivery. Maternal smoking was categorized into two categories: ‘never smoked’ and ‘smoking during pregnancy.’ Covariates signifying autoimmune diseases and gestational diabetes were categorized into two categories: ‘Yes’ and ‘No.’

We adjusted for technical covariate, i.e., Illumina plate factor. It should be noted that the cellular DNA source of saliva is heterogeneous. While there is much literature dealing with saliva cell type composition in infants, children and adults [78, 93, 99], to date, there are no studies indicating the cell mixture composition of the saliva of the newborn. Housemann et al., in 2014 [100], introduced the reference-free cell type method to estimate cell types in tissues such as saliva, placenta and adipose tissue [100], which is closely related to surrogate variable analysis (SVA) [101]. We have therefore used SVA directly to estimate for all the unobserved variability including cell types. It has been shown that using SVA increases the biological accuracy and reproducibility by identifying the sources of heterogeneity and correctly accounting for them in the analysis [101]. Three surrogate variables that were generated in the quality control step, using the *SVA* package, were also used as covariates in the main model.

### Data analysis

**Fig. 7:**
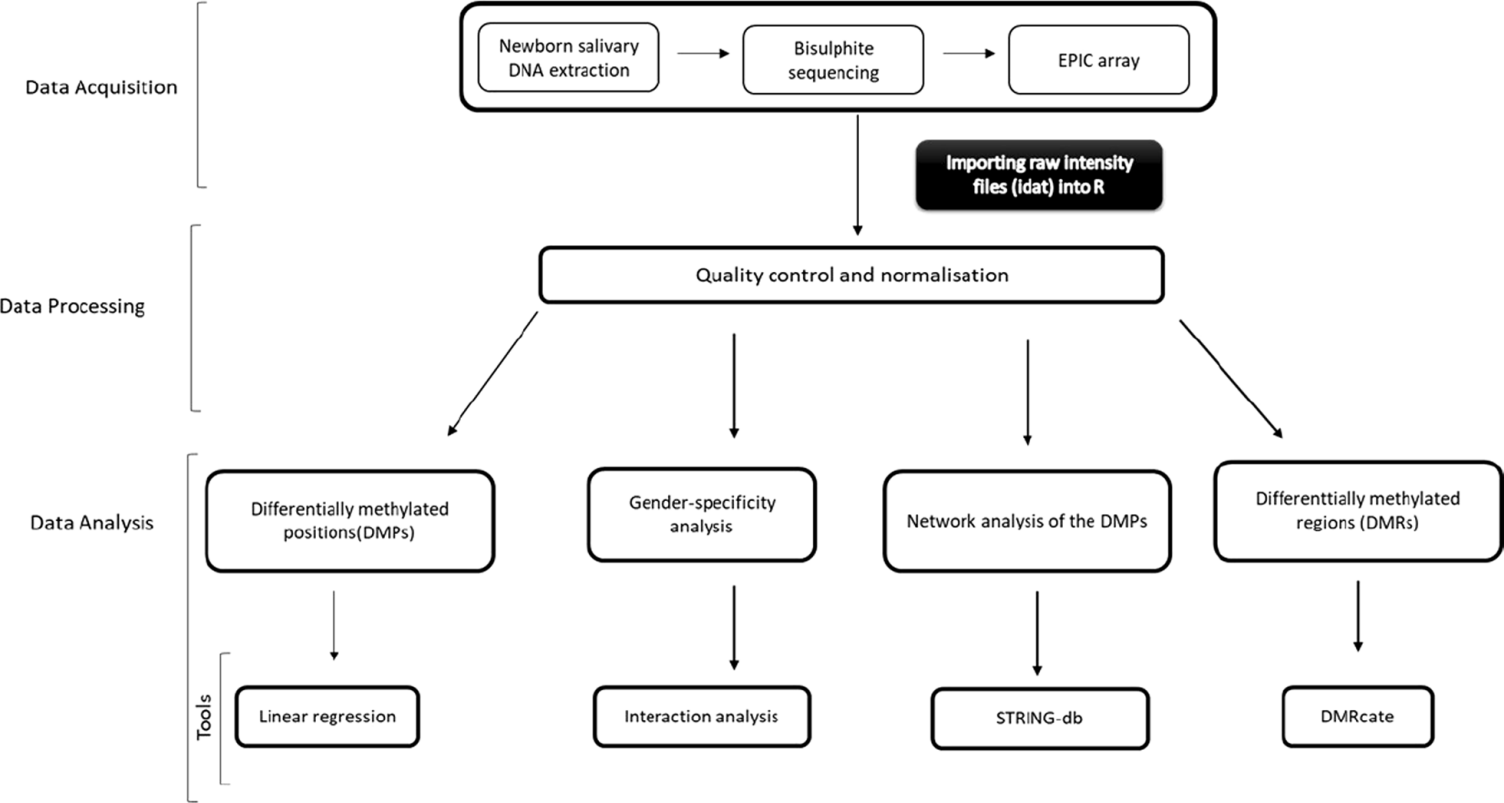
Overall methodological study design. Illumina measured salivary DNA methylation using the EPIC microarray platform. The raw data were processed and quality-controlled using array-specific algorithms in R studio. Data visualization and statistical analysis identified relevant associations and derived a list of differentially methylated positions and regions

We conducted a series of analyses on the genome-wide microarray data, with each technique designed to capture potentially different patterns of DNA methylation. The first analysis conducted was EWAS analysis for the DMPs (CpG or site-by-site regression analysis), which analyzed each CpG site individually. Second, we performed a sex interaction EWAS analysis for the DMPs to compare methylation patterns in terms of sex. The Illumina database (‘IlluminaHumanMethylationEPICanno.ilm10b4.hg19’) was primarily used for identifying gene annotations for the significant hits. The UCSC genome browser was used to verify genes identified with Illumina database and, where genes were missing in the Illumina database, was searched to augment genes within 50 kb of the CpG site. Third, the biological exploration and network analysis for each CpG annotating to specific genes was conducted using the online software STRING-DB [25] and SFARI [26]. Fourth, we conducted DMR analysis (or regional analysis), which captures an average pattern of DNA methylation among neighboring sites. Figure 7 shows a summary of the methodological study design.

#### Differentially methylated positions (DMPs) analysis

Each CpG site or DMP was separately tested for association with exposure to stress. Three sets of EWAS analyses were run to identify CpG sites associated with either PSS, PDQ, FSI or cortisol. All the statistical analyses were done in R version 3.5.2. We used the following linear regression model in ‘limma’ R package to test for DMPs:—DNA methylation ~ Stress measures + Newborn sex + Gestational age + Smoking + Autoimmune diseases + gestational diabetes + Illumina plate factor + SVAs. We visualized the epigenome-wide associations study results using Manhattan plots and quantile–quantile (QQ plots). Genomic inflation factor was calculated for each association. We corrected the *p* values for inflation if lambda was above 1.1, using a Bayesian method for estimation of empirical null distribution as implemented in R/Bioconductor package ‘bacon’ [102].

#### Sex interaction analysis

Sex interaction analysis was performed in the FELICITy cohort for each CpG/DMP site association with the stress measures. The model was identical to the adjusted model, but with a ‘Sex * methylation’ interaction term (male as a reference sex). Statistical significance threshold was set at FDR < 0.05.

#### Exploratory analysis of DMPs

To investigate whether specific biological processes and networks are overrepresented in our EWAS results, network analyses were performed for the DMPs using STRING-db. The protein encoding genes that were annotated to significant DMPs were analyzed using STRING-DB. STRING-DB is an unsupervised statistical network analysis database that has known proteins and their physical and functional interaction networks [25]. We used the KEGG database in STRING-DB to explore whether annotated genes have been related to neurobiological and neuronal processes or diseases. We also used the SFARI gene database [26] to extract information for the genes annotated to CpGs specific to ASD [27].

#### Differentially methylated regions (DMRs) analysis

DMRs were initially identified using the Bioconductor DMRcate package [103] and verified using comb-p [104]. These packages are consistently reported to have the best sensitivity and highest control of false positive rate when compared to other DMR tools [105]. A significant DMR can be detected even if there is no genome-wide significant DMP in the region. DMRcate identifies DMRs from the tunable kernel smoothing process of association signals [103].

DMRcate was used on the results of the limma analysis to test for DMRs. The parameters for DMRcate (lambda = 1000, C = 2) were set, and a FDR cutoff of 0.05 was used to determine significance. Further Comb-p was used to verify DMRs identified by DMRcate. For comb-p, identified DMRs consisting of at least two probes and having a Sidak-corrected *p* value < 0.05 were considered statistically significant [104]. DMRs were annotated to gene symbols according to genome assembly (hg19). *p* value for each DMR was adjusted for multiple testing with Sidak correction method as implemented by default in the ‘comb-p’ tool.

## Supplementary Information

**Additional file 1. S1.** Uncorrected and BACON corrected Quantile-quantile plot. **Additional file S2**. Enrollment flowchart for FELICITy study.

## Data Availability

The datasets supporting these findings are not publicly available. Instead, the datasets used and/or analyzed for the current study are available from the corresponding author on reasonable request and after Institutional Review Board review and approval. Scripts used in data processing and statistical analyses have been made publicly accessible at https://ascgitlab.helmholtz-muenchen.de/ritika.sharma/felicity-project/-/tree/master/

## Abbreviations

ADHD: Attention-deficit hyperactivity disorder
ANS: Autonomic nervous system
*ARL4D*: ADP-ribosylation factor 4D
ASD: Autism spectrum disorder
CNS: Central nervous system
*CSMD1*: CUB and Sushi multiple domains 1
CTG: Cardiotocography
*DAXX*: Death-associated protein 6
DMPs: DNA-methylated positions
DMR: DNA-methylated regions
ECG: Electrocardiogram
EWAS: Epigenome-wide association study
FHR: Fetal heart rate
FSI: Fetal stress index
HPA axis: Hypothalamic–pituitary–adrenal axis
MHR: Maternal heart rate
PDQ: Prenatal Distress Questionnaire
PRSA: Phase-rectified signal averaging
PS: Prenatal stress
PSS: Perceived Stress Scale
PTSD: Post-traumatic stress disorder
QQ: Quantile–quantile
SNPs: Single-nucleotide polymorphism
SV: Surrogate variables
TaECG: Transabdominal electrocardiography
*TOMM20*: Translocase of outer mitochondrial membrane 20
UCSC: University of California Santa Cruz
*YAP1*: Yes1-regulated transcription factor
