# Supplementary material for: Maternal–fetal stress and DNA methylation signatures in neonatal saliva: an epigenome-wide association study
Source: Clin Epigenetics. 2022 Jul 14;14:87. doi: 10.1186/s13148-022-01310-x (PMC9281078; doi:10.1186/s13148-022-01310-x)
Supplement: Supplementary file 1 — Additional file 1. S1. Uncorrected and BACON corrected Quantile-quantile plot. Additional file S2. Enrollment flowchart for FELICITy study. [file 13148_2022_1310_MOESM1_ESM.docx]

**Additional File S1**

**
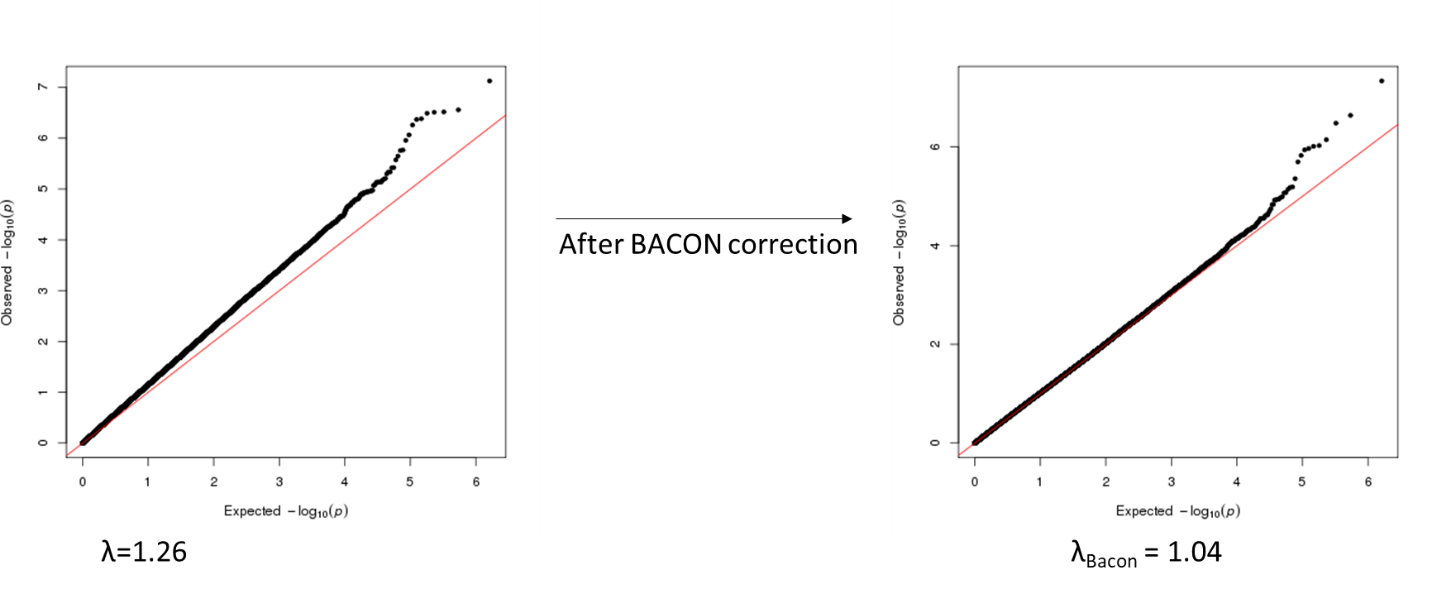
**

**Fig S1: Extended Q-Q plot of salivary DNA methylation associated with PDQ.**

Quantile-quantile (QQ) plots of observed and expected distributions of p-values of the association between salivary DNA methylation and PDQ. Lambda (λ) is the genomic inflation factor, and λ_Bacon_ is the genomic inflation factor after correction for inflation, estimated using the method of van Iterson et al. (2017) (102), implemented using the *bacon* R package.

**Additional File S2**


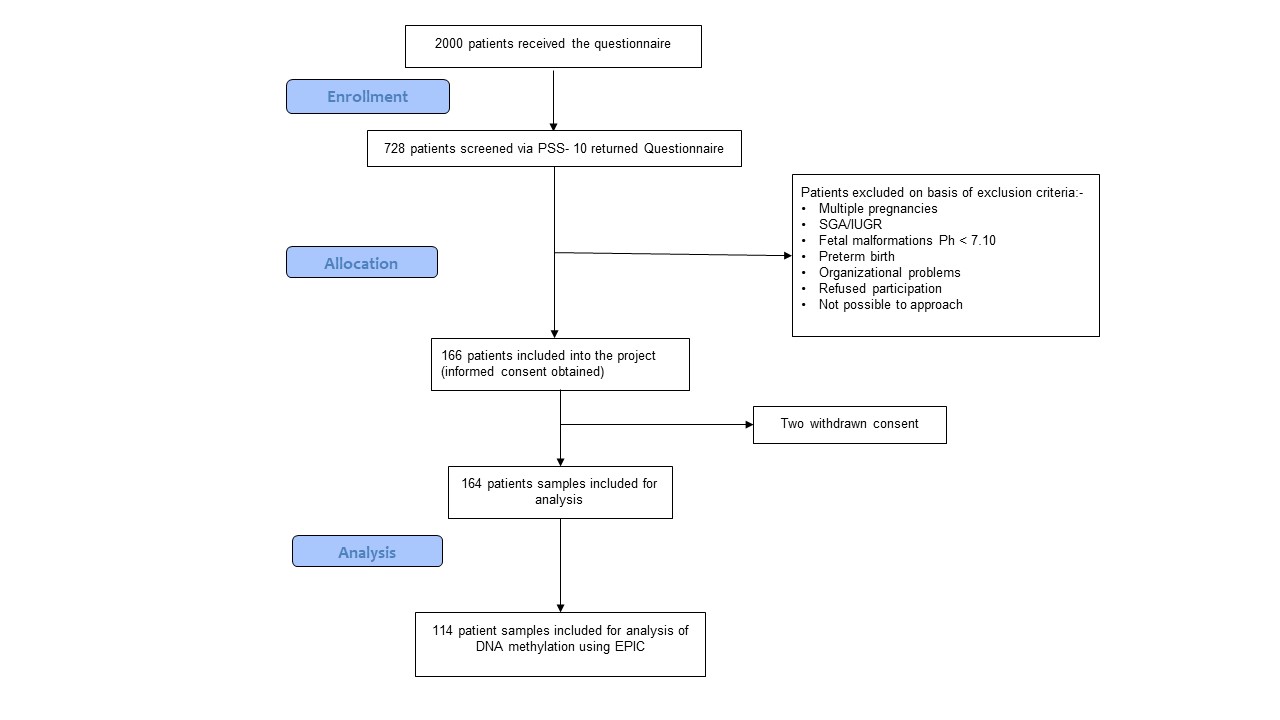


**Fig S2: Enrollment flowchart for FELICITy Study**
